# Gut microbiome and blood biomarkers reveal differential responses to aerobic and anaerobic exercise in collegiate men of diverse training backgrounds

**DOI:** 10.1038/s41598-025-99485-9

**Published:** 2025-05-08

**Authors:** Kinga Humińska-Lisowska, Monika Michałowska-Sawczyn, Tomasz Kosciolek, Paweł P. Łabaj, Andrzej Kochanowicz, Jan Mieszkowski, Patrizia Proia, Paweł Cięszczyk, Kinga Zielińska

**Affiliations:** 1 Faculty of Physical Culture, Gdansk University of Physical Education and Sport, Gdansk, Poland; 2 Sport and Exercise Sciences Research Unit, Department of Psychology, Educational Science and Human Movement, University of Palermo, Palermo, Italy; 3 Sano Centre for Computational Medicine, Krakow, Poland; 4 Malopolska Centre of Biotechnology, Jagiellonian University, Krakow, Poland; 5 Faculty of Health Sciences, University of Lomza, Lomza, Poland

**Keywords:** Gut Microbiome, Aerobic and anaerobic exercise, Inflammatory biomarkers, Metabolic adaptation, Microbiome‒host crosstalk, Athletic performance, Microbiology, Physiology, Biomarkers

## Abstract

The gut microbiome influences physiological responses to exercise by modulating inflammatory markers and metabolite production. Athletes typically exhibit greater microbial diversity, which may be associated with improved performance, but the mechanisms linking different exercise modalities to the gut microbiome are not fully understood. In this study, blood and stool samples were collected from endurance athletes, strength athletes, and non-athletic controls performing two maximal exercise tests (the anaerobic Wingate test and the aerobic Bruce Treadmill Test) to integrate serum biomarker data with gut bacterial metagenomic profiles. While most biochemical markers showed similar post-exercise trends across groups, SPARC (secreted protein acidic and rich in cysteine) and adiponectin levels showed modality-specific responses. Strength-trained participants showed unique microbiome-biomarker associations after the Wingate test. In addition, baseline enrichment of certain bacterial taxa, including *Clostridium phoceensis* and *Catenibacterium spp*., correlated with reduced Bruce Treadmill test response in strength-trained individuals. These findings, while requiring further validation, indicate the complex interplay between exercise type, training background, and the gut microbiome, and suggest that specific microbial species may help shape recovery and adaptation.

**Supplementary Information:**

The online version contains supplementary material available at 10.1038/s41598-025-99485-9.

## Introduction

The human gut microbiome is a community of microorganisms, including bacteria, that interact with each other and with their host in a specific ecological niche. The microbiome is a dynamic community that displays a wide range of functions and produces various metabolites^1^. The human gut microbiome is influenced by various host factors, such as sleep^2^, diet^3^, age^4^ and exercise^5^. Owing to its ability to adapt to different environmental conditions, it has been extensively studied in relation to sports performance^6^.

Over 10 years ago, it was already acknowledged that athletes have a more diverse and richer gut microbiome compared to non-athletes^7^. This diversity is linked to improved metabolic health, enhanced immunity, and better overall performance. While a number of training-beneficial species and mechanisms through which the gut microbiome interacts with the host in the context of sports are known, many findings remain ambiguous^8^.

Exercise, both aerobic and anaerobic, induces specific physiological responses and adaptations reflected in changes in blood serum markers. Aerobic exercise, characterized by sustained, moderate-intensity activities that rely on oxidative metabolism, improves cardiovascular efficiency and endurance performance^9^. Anaerobic exercise involves high-intensity, short-duration activities that rely on anaerobic energy pathways and result in increased muscle strength and power^10^. These training modalities influence the secretion of various proteins affecting human organs, including inflammatory markers, hormones, and cytokines^11^. The intensity, direction, and rate of these responses depend on many factors and do not always result in beneficial changes in body structure. Prolonged exercise duration and intensity contribute to increased oxidative stress, which can lead to a greater risk of injury and dysfunction of working tissues^12^.

The gut microbiome plays a significant role in modulating these physiological responses through its interactions with inflammatory markers. Specific gut bacteria can influence systemic inflammation by producing metabolites such as short-chain fatty acids (SCFAs), which have anti-inflammatory properties^13^. Conversely, inflammation can alter the gut microbiome composition, creating a bidirectional relationship between the microbiome and the host immune system^14^. Understanding these interactions is crucial, as they may impact recovery processes and overall athletic performance.

To capture a broad spectrum of inflammatory, metabolic, and tissue remodeling processes potentially influenced by gut microbiome-host interactions, we selected a panel of serum markers reflecting key pathways relevant to exercise physiology. Specifically, adiponectin, leptin, and resistin are key adipokines involved in energy balance, insulin sensitivity, and metabolic regulation^15^. Interleukin (IL)-1 alpha, IL-6, IL-10, IL-15, oncostatin M (OSM), and leukemia inhibitory factor (LIF) are central cytokines that drive inflammation, immune signaling, and muscle repair^16–18^. Meanwhile, SPARC (secreted protein acidic and rich in cysteine) and follistatin-like 1 (FSTL1) contribute to tissue remodeling and cardiovascular function^19,20^, while TIMP-1 (tissue inhibitor of metalloproteinases 1) is integral to extracellular matrix remodeling during muscle stress^21^. Finally, the transferrin receptor (TfR) reflects iron metabolism and erythropoiesis, both of which are essential for maintaining oxygen transport in active individuals^22^. By measuring these markers, we aimed to provide a broad snapshot of how different exercise modalities might shape systemic responses in conjunction with the gut microbiome, thereby allowing for a more integrative understanding of exercise-induced physiological changes.

In our previous study, we sought to evaluate the gut microbiome and its response to acute exercise interventions performed at maximal intensity^23^. We found that individuals with an athlete-level VO_2max_ (VO_2_max = 61.3 +- 7.6 ml/kg/min^24^, Supplementary Table S1) experienced an enrichment of *Bifidobacterium longum*, a probiotic species commonly represented in commercial products, as well as the short-chain fatty acid producer *Roseburia inulinivorans*, after aerobic effort. In addition, we identified correlations between maximal power output and the SCFA producers *Eubacterium rectale*, *Blautia wexlerae* and *Intestinimonas timonensis*. Various individual responses were observed, however, suggesting complex microbiome interactions.

Here, motivated by numerous studies indicating significant changes in inflammatory markers induced by maximal exercise, we integrated blood serum marker data with gut bacterial metagenomic data (originally analyzed in our previous study^23^). We began by outlining markers that changed independently of exercise modality and training background. Afterwards, we examined markers that exhibited similar trends across all participant groups but varied depending on the exercise modality. Finally, we identified markers differentiating participants based on their training background. By exploring microbial associations with blood markers, we aimed to uncover potential microbial behaviors that could influence individual responses to training interventions in the control, endurance, and strength groups. This approach allowed us to identify baseline microbiome features that could predispose certain individuals to an excessive stress response following exercise.

Therefore, the primary purpose of this study was to determine whether different exercise modalities (anaerobic vs. aerobic) and training backgrounds (control, endurance, strength) lead to different responses in selected blood biomarkers and the gut microbiome. We hypothesized that each exercise modality and training background would produce unique patterns in serum marker levels and microbial composition, reflecting distinct host-microbiome interactions in response to acute maximal exercise. While our findings are preliminary, they suggest that specific gut microbial compositions might modulate inflammatory and metabolic adaptations to training, warranting further investigation into their potential role in exercise-induced physiological responses.

## Materials and methods

### Experimental overview

This study is an extension of our previous investigation^23^, which aimed to evaluate the gut microbiome and its response to different exercise modalities in different athletic populations. This study integrates biochemical analyses and advanced statistical and bioinformatic methods to further understand the interplay among exercise, the microbiome and biochemical markers.

This interventional, case‒control, repeated-measures study focused on the gut microbiome composition and biochemical responses before and after two different exercise tests: (1) a repeated 30-s all-out Wingate test (WT) and (2) the Bruce Treadmill Test. The fitness tests were performed at the University of Physical Education in Gdansk (Gdansk, Poland) in October 2018, following the same protocol as previously described^23^. The Wingate and Bruce treadmill tests were separated by a 14-day break, during which time the participants maintained their regular physical activities and training routines.

### Participants

A total of 52 male participants were recruited and divided into three groups: endurance athletes (*n* = 15), strength athletes (*n* = 16), and a control group (*n* = 21). Endurance athletes had at least 5 years of training in race walking, long-distance running, or ski running, whereas strength athletes had a similar duration of training in weightlifting, powerlifting, or bodybuilding. The control group consisted of physically active men who did not participate in organized training but were involved in recreational sports and attended physical education classes at the University of Physical Education and Sport in Gdansk, Poland. There were no statistically significant morphological differences between the control, endurance, and strength groups. The inclusion and exclusion criteria were consistent with those of our previous study^23^.

The study protocols were conducted in accordance with the Declaration of Helsinki and approved by the Bioethics Committee for Clinical Research of the Regional Medical Society in Gdansk (KB-27/18). All participants provided written informed consent. Personal data were encoded to ensure anonymity and privacy.

### Measurement of anaerobic and aerobic fitness

The following procedures were described in our previous study^23^. In brief, the Wingate Test (WT) - a short-term, all-out cycling test recognized as a standard measure of anaerobic power and capacity was performed on a Monark 894E friction-loaded cycle ergometer (Monark 894E, Peak Bike from Sweden) after a 5 min warm-up at 60 rpm (1 W/kg). Participants performed two 30-second bouts of maximal effort pedalling against a resistance load of 75 g/kg body mass, with a 30-second rest between bouts, as recommended by Bar-Or^25^. The performance measures included peak power, relative peak power, mean power, and relative mean power. The aerobic Bruce Treadmill Test - a widely used graded treadmill test designed to measure maximal oxygen uptake (VO_2_max) and assess cardiovascular fitness - was administered on an H/P/Cosmos electric treadmill^26^. Following a 5-minute warm-up at 60% HR max, participants progressed through 10 stages of increasing difficulty, starting at a 10% incline and 2.7 km/h and ending at a 28% incline and 12.07 km/h. The test continued until voluntary exhaustion or an inability to maintain the required treadmill speed and grade. Maximal oxygen uptake was measured via a Quark CPET pulmonary gas exchange analyzer.

### Sample collection and measurements of selected markers

The procedures were adapted from our previous studies^23,27^. Blood samples (9 mL) were collected at five time points: immediately before, immediately after, 2 h, 6 h, and 24 h after each test. Venous blood samples were collected in Sarstedt S-Monovette tubes (S-Monovette^®^, Sarstedt AG&Co, Nümbrecht, Germany) equipped with a coagulation accelerator for serum separation. The serum was processed according to standard laboratory protocols, aliquoted into 500 µL, and stored at -80 °C until further analysis (up to 6 months). The following markers were measured: adiponectin, follistatin-like 1 (FSTL1), interleukin (IL)-1 alpha, IL-6, IL-10, IL-15, leptin, leukemia inhibitory factor (LIF), oncostatin M (OSM), resistin, secreted protein acidic and rich in cysteine (SPARC), metalloproteinase inhibitor 1 (TIMP-1), and transferrin receptor (TfR). Analyses were performed via a MAGPIX fluorescence detection system (Luminex Corp., Austin, TX, USA) with Luminex assays (Luminex Corp.; Human Magnetic Luminex Assay (13-plex)).

In addition, fresh stool samples were collected from all participants at three time points: time point zero (T0: W0 for the Wingate test or B0 for the Bruce treadmill test), the morning after an overnight fast of approximately 8 h before the exercise test; time point one (T1: W1 or B1), the same day after the exercise test; and time point two (T2: W2 or B2), the next morning on an empty stomach. The samples were placed in stool containers, immediately placed in thermal bags with cooling inserts, and kept refrigerated for a short time. The samples were delivered to the laboratory within a few hours and immediately snap frozen at -80 °C.

### Metagenomic sample Preparation

The procedures for sample preparation, DNA isolation, quantification, and library preparation followed the methods described in our previous publication^23^. Briefly, DNA was extracted from 200 mg of the frozen stool samples via a modified NucleoSpin^®^ DNA Stool Kit (Macherey-Nagel, Germany) via physical, mechanical, and chemical lysis. The DNA concentration and purity were measured via a NanoDrop spectrophotometer and a Qubit fluorometer. Library preparation followed the KAPA HyperPlus protocol, and sequencing was performed on an Illumina NovaSeq platform, generating approximately 22 million 150 bp paired-end reads per library. The raw fastq files are openly available in the European Nucleotide Archive (ENA) at https://www.ebi.ac.uk/ena/, reference number PRJEB60692.

### Statistical and bioinformatic analysis

The raw fastq sequences were subjected to quality control via TrimGalore^28^. Taxonomic and functional profiles were calculated via MetaPhlAn 4.0 and HumanN 3.7^29^. The differences between blood serum marker levels at consecutive time points were evaluated via repeated-measures ANOVA with Benjamini-Hochberg correction for multiple testing (Python’s *statsmodels.stats.multitest* package). The correlations of the blood markers and metagenomic features were performed in two ways: (a) correlations at single timepoints and (b) changes in blood markers and metagenomic features between two timepoints, which were correlated via Spearman with Benjamini‒Hochberg correction for multiple testing^30^. Finally, we used Maaslin2 with default settings to perform differential enrichment analysis of species and pathways distinguishing the baseline microbiomes of the responders and nonresponders to the Bruce intervention^31^.

## Results

### Most biochemical changes are independent of training background and exercise modality

A previously published cohort consisting of 52 male participants was divided into endurance (*n* = 15), strength (*n* = 16) and control (*n* = 21) groups on the basis of training background^23^. All participants were subjected to two maximal effort interventions: an anaerobic-focused Wingate test and an aerobic-focused Bruce treadmill test. Stool samples were collected before and twice after each intervention. For the purpose of this study, we included biochemical parameter collection timepoints that approximated the time of stool collection (there were three stool and five biochemical collections in total).

**Fig. 1:**
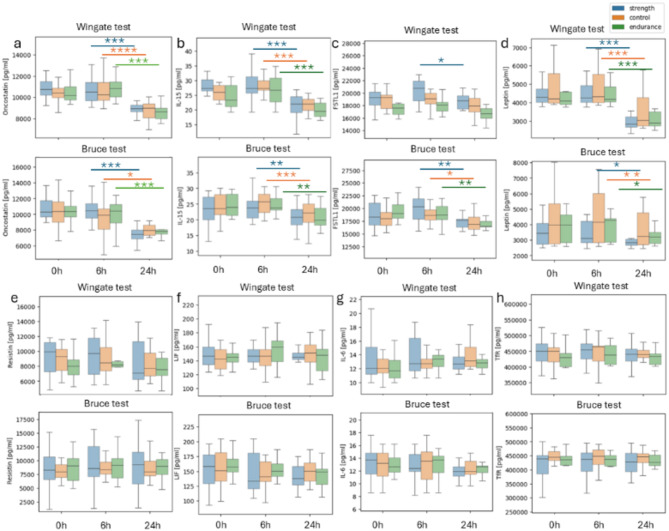
Post-exercise changes in biochemical markers in all groups following the Wingate (top) and Bruce (bottom) tests. Most biochemical parameters show similar trends in all groups after the Wingate (top) and Bruce (bottom) tests. Blue: strength, orange: control, green: endurance. (**a**) oncostatin, (**b**) IL-15 (interleukin 15), (**c**) FSTL1 (Follistatin-like 1), (**d**) leptin, (**e**) resistin, (**f**) LIF—leukemia inhibitory factor, (**g**) IL-6 (interleukin 6), (**h**) TfR (transferrin receptor); *p < 0.05, **p < 0.01, ***p < 0.001.

Most biochemical parameters showed similar trends in all groups after the Wingate (Fig. 1 top) and Bruce (Fig. 1 bottom) interventions.

Oncostatin M (OSM) and IL-15 levels (Fig. 1a, b) significantly decreased after both the Wingate and Bruce tests in all groups (endurance, strength, and control) at 24 h post exercise. Follistatin-like 1 (FSTL1) levels (Fig. 1c) slightly increased 6 h after the Wingate test and significantly decreased 24 h after the Wingate test (p adjusted < 0.05). After the Bruce treadmill test, FSTL1 levels also slightly increased at 6 h, followed by a statistically significant decrease at 24 h in all groups (p adjusted < 0.05), suggesting a consistent pattern of initial increase and subsequent decrease in response to both types of exercise. A statistically significant decrease in leptin levels was observed 24 h after the Wingate test in all groups (Fig. 1d, p adjusted < 0.001). Conversely, the levels of leukemia inhibitory factor (LIF), resistin, interleukin-6 (IL-6), and transferrin receptor (TfR) (Fig. 1e-h) remained stable in both the Wingate and Bruce tests. These markers did not significantly change, indicating that they were not significantly affected by the exercise interventions. In conclusion, even if significant changes were observed only after one test, they would follow a similar pattern after the other intervention. Therefore, the aforementioned parameters were not associated with the differential responses to exercise modalities among the groups. Further statistics can be found in Supplementary Table S2.

### Nucleotide recycling as a modulator of responses to different training efforts

**Fig. 2:**
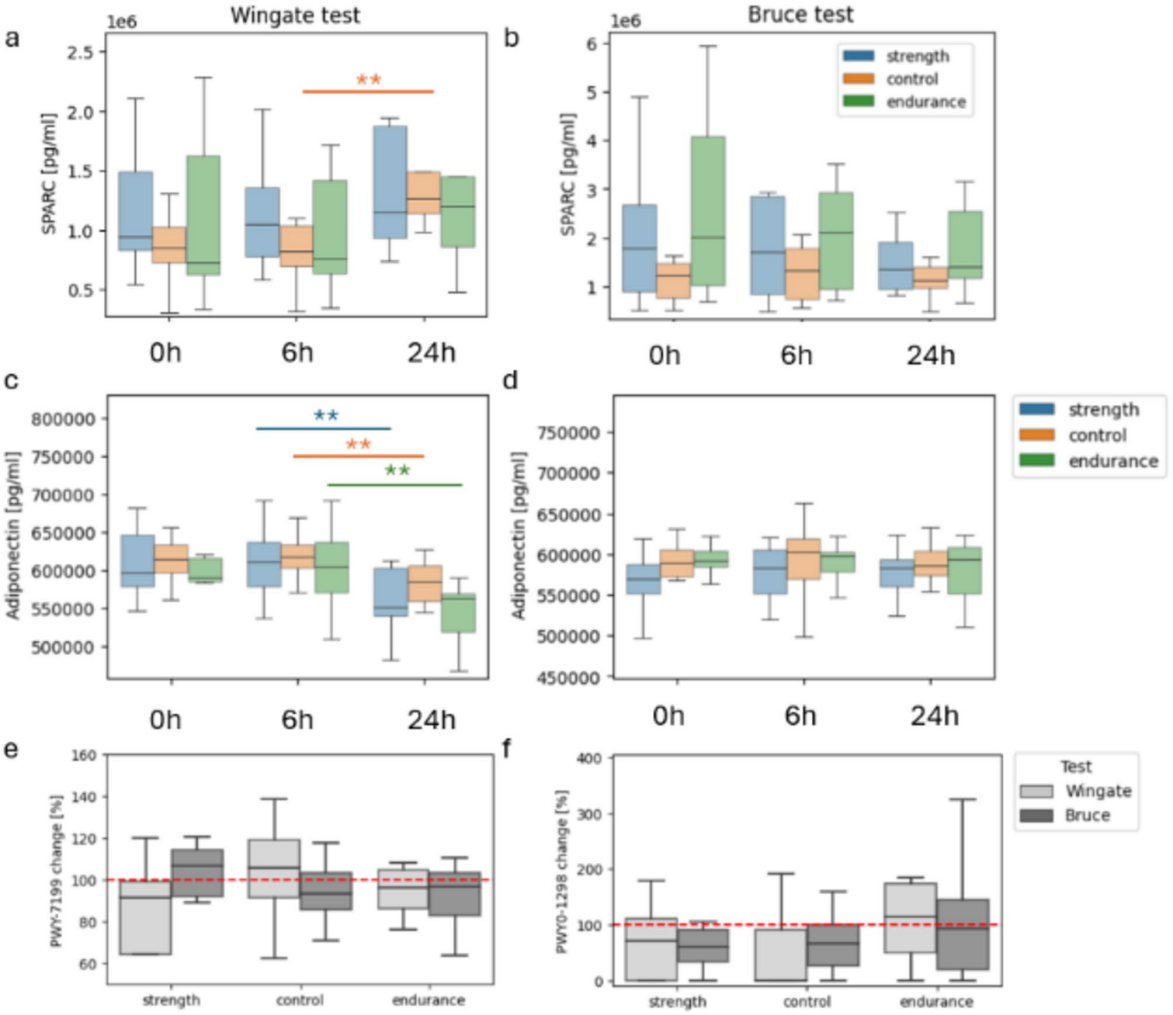
Distinct trends in the SPARC, adiponectin and pyrimidine pathways following the Wingate and Bruce tests. Different secreted *protein* acidic and rich in cysteine (SPARC) trends between timepoints before exercise, 6 h after exercise and 24 h after the Wingate (**a**) and Bruce (**b**) tests. Different Adiponectin trends between timepoints after the Wingate (**c**) and Bruce (**d**) tests. A relative abundance changes between timepoints 6 h and 24 h after the Wingate (**e**) and Bruce (**f**) interventions of the *PWY-7199: pyrimidine deoxyribonucleosides salvage pathway* and *PWY0-1298: superpathway of pyrimidine deoxyribonucleosides degradation*. A % change of 100 indicates no change; *p adjusted < 0.05, **p adjusted < 0.01.

SPARC and adiponectin were the only biochemical parameters that behaved differently as a result of the interventions and were similar in every group (Fig. 2a-d). SPARC levels significantly increased only in the control group 24 h after the Wingate test (*p* < 0.05). Although there was an insignificant trend in the endurance and strength groups, it was in the same direction as that in the control. No statistically significant changes in SPARC levels were observed after the Bruce test; however, the trend decreased. On the other hand, adiponectin significantly decreased between 6 h and 24 h after the Wingate testin all groups,. It remained steady throughout and after the Bruce intervention. The divergent responses of SPARC and adiponectin could be attributed to the different physiological demands and stress responses induced by the Wingate and Bruce tests.

We identified distinct statistically significant correlations of microbiome feature changes with changes in adiponectin and SPARC between 6 h and 24 h after the Wingate and Bruce interventions. The Wingate intervention was correlated with *PWY0-1477: ethanolamine utilization* with both SPARC (Spearman correlation of 0.85, *p* < 0.001, p adjust < 0.2) and adiponectin in the strength group (*r* = 0.90, *p* < 0.001, p adjust = 0.05). Associated only with SPARC were *Blautia massiliensis* (*r* = 0.83, *p* < 0.01, p adjust < 0.2) and the *PWY-7199: pyrimidine deoxyribonucleoside salvage pathway* (*r* = 0.83, *p* < 0.01, p adjust < 0.2). Interestingly, the only association in the control group was also linked to pyrimidine deoxyribonucleosides; however, in this case, it was its degradation (*PWY0–1298: superpathway of pyrimidine deoxyribonucleotide degradation* correlated with adiponectin, *r*=-0.92, *p* < 0.0001, p adjust < 0.01). A further investigation revealed associations of the pathways after the Bruce test as well: *the pyrimidine deoxyribonucleotide salvage pathway* was correlated with TIMP1 in the endurance group (*r* = 0.78, *p* < 0.01, p adjust < 0.1), and *the superpathway of pyrimidine deoxyribonucleotide degradation* was strongly correlated with IL-15 (*r*=-0.95, *p* < 0.0001, p adjust = 0.02). A comparison of the changes in the relative abundances of the pathways at 6 h and 24 h after the Wingate and Bruce interventions revealed nonsignificant but notable differences between the interventions and the other groups (Fig. 2e, f). These results indicate a complex response of the gut microbiome to intense athletic efforts via the mechanisms of nucleotide recycling.

After the Bruce intervention, adiponectin was correlated with *PWY3O-4107: NAD salvage pathway V (PNC V cycle) and PWY-4041: &gamma;-glutamyl cycle* (Spearman correlations of -0.92 and − 0.90, respectively, both *p* < 0.001 and p adjust < 0.01). We identified an association of SPARC with *PWY0-1241: ADP-L-glycero-&beta; D-manno-heptose biosynthesis* in the control (*r*=-0.80, *p* < 0.01, p adjust < 0.2). In contrast, no associations with blood markers after the Bruce test were found in the strength group.

### Background training shapes the microbiome response to exercise

#### Wingate test

The next step involved an analysis of biochemical parameters that had different trends in the training groups after the Wingate intervention. Correlations with microbiome feature abundances at 6 h and 24 h revealed various associations, mainly in the strength group (Table 1). This confirmed our hypothesis that exercise modality would impact individuals adapted to it the most. We identified two pathways in the strength group that were positively correlated with multiple blood markers. *PWY-5989: stearate biosynthesis II* was associated with IL-15, Il-1a and Il-10, whereas *PWY0-1477: ethanolamine utilization* with adiponectin and SPARC (aforementioned in the previous section). In addition, we identified a number of blood marker connections with *Blautia spp*., *Lactococcus lactis*, *Alistipes onderdonkii*, *Sutterella wasdworthensis* and *Roseburia intestinalis*, as well as *PWY-6123: inosine-5’-phosphate biosynthesis* I and *GLUDEG-I-PWY: GABA shunt pathways*.

**Table 1:** Spearman correlations of the Microbiome and parameter changes between timepoints 6 h and 24 h after wingate intervention. Only correlations with significant p-values (< 0.05) are included.

Group	Parameter	Microbiome feature	Spearman correlation	Adjusted *p* value
Control	Adiponectin [pg/ml]	PWY0-1298: superpathway of pyrimidine deoxyribonucleosides degradation	− 0.92	**< 0.01**
	TIMP1 [pg/ml]	Parasutterella excrementihominis	− 0.87	**0.04**
	Resistin [pg/ml]	PWY-6305: superpathway of putrescine biosynthesis	0.86	0.06
Strength	Adiponectin [pg/ml]	PWY0-1477: ethanolamine utilization	0.90	0.05
	IL-15 [pg/ml]	PWY-5989: stearate biosynthesis II (bacteria and plants)	0.90	**0.03**
	IL-1a [pg/ml]	PWY-5989: stearate biosynthesis II (bacteria and plants)	0.90	**0.04**
	Resistin [pg/ml]	Blautia glucerasea	0.88	0.10
	Il-10 [pg/ml]	PWY-6123: inosine-5’-phosphate biosynthesis I	0.86	0.10
	Il-10 [pg/ml]	PWY-5989: stearate biosynthesis II (bacteria and plants)	0.86	0.10
	FSTL1 [pg/ml]	Lactococcus lactis	0.86	0.20
	SPARC [pg/ml]	PWY0-1477: ethanolamine utilization	0.85	0.18
	SPARC [pg/ml]	Blautia massiliensis	0.83	0.18
	Resistin [pg/ml]	GGB9758 SGB15368	0.82	0.18
	Resistin [pg/ml]	Alistipes onderdonkii	0.82	0.18
	SPARC [pg/ml]	PWY-7199: pyrimidine deoxyribonucleosides salvage	0.82	0.18
	Resistin [pg/ml]	Candidatus Cibiobacter qucibialis	0.82	0.18
	Resistin [pg/ml]	Ruminococcaceae unclassified SGB15234	0.82	0.19
	Il-10 [pg/ml]	GLUDEG-I-PWY: GABA shunt	− 0.85	0.13
	Resistin [pg/ml]	Sutterella wadsworthensis	− 0.86	0.16
	LIF [pg/ml]	Roseburia intestinalis	− 0.87	0.16
Endurance	LIF [pg/ml]	Faecalibacterium SGB15346	0.93	0.07
	TIMP1 [pg/ml]	Bacteroides uniformis	− 0.92	0.15
	TIMP1 [pg/ml]	Bacteroides ovatus	− 0.90	0.15
	TIMP1 [pg/ml]	PWY0-1261: anhydromuropeptides recycling I	− 0.90	0.15

TIMP1 (tissue inhibitor of metalloproteinases), IL-1a (interleukin 1a), IL-5 (interleukin 5), IL-10 (interleukin 10), IL-15 (interleukin 15), LIF (leukemia inhibitory factor), FSTL1 (follistatin-like 1), and SPARC (secreted protein acidic and cysteine rich). Significant correlations (p-value < 0.05) were observed in all groups, with the strength group showing the most notable associations. Pathways such as stearate biosynthesis II and ethanolamine utilization were positively correlated with multiple blood markers, including IL-15, IL-1a, IL-10, adiponectin, and SPARC. Additionally, several microbiome features, including *Blautia spp.*, *Lactococcus lactis*, *Alistipes onderdonkii*, *Sutterella wadsworthensis*, and *Roseburia intestinalis*, were significantly associated with resistin and other inflammatory markers. These results highlight the complex interplay between microbiome features and blood markers following the Wingate intervention.

Significant values are in bold.

**Fig. 3:**
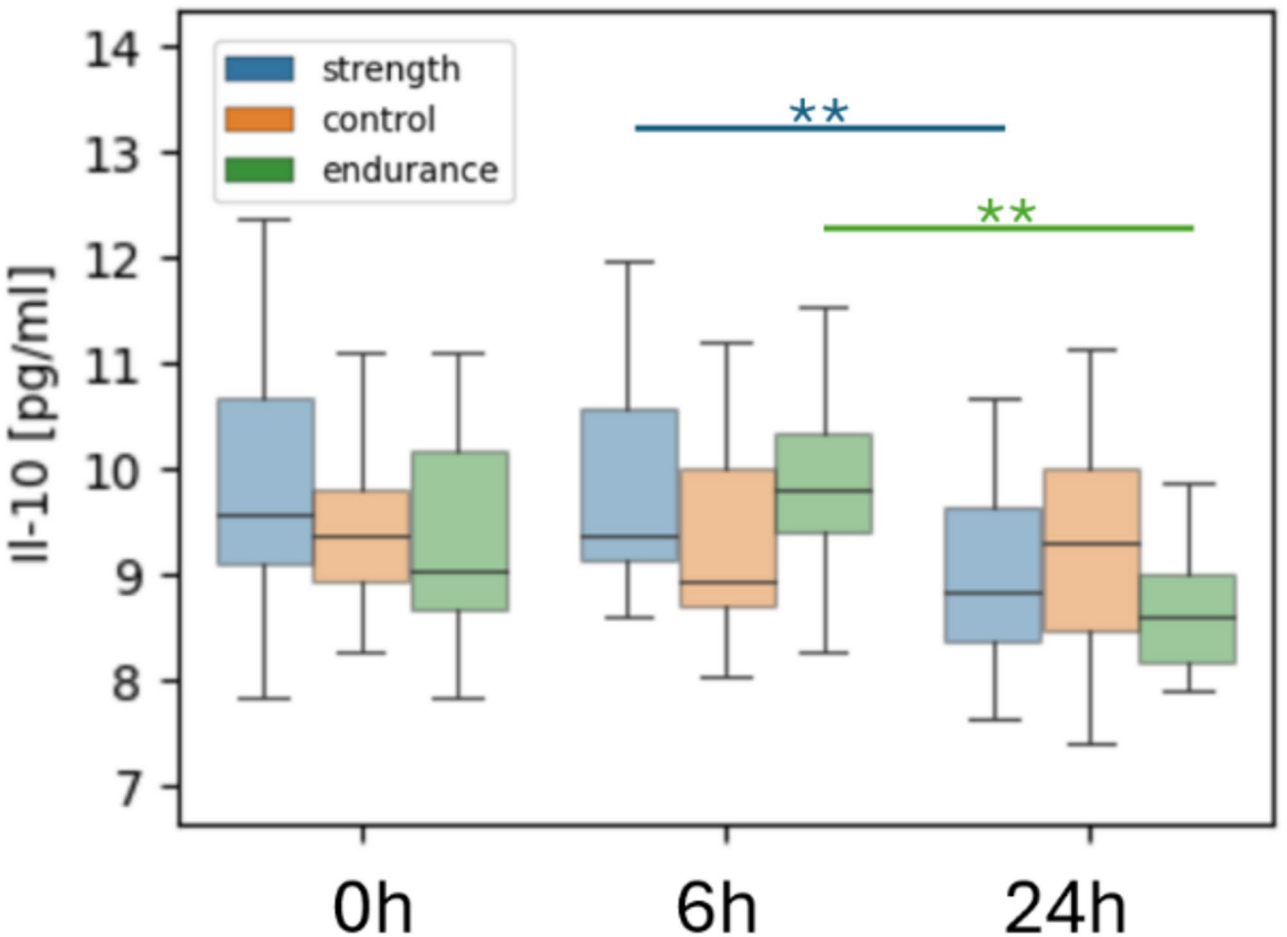
Differences in IL-10 levels between training groups after the Wingate test. Average interleukin 10 (IL-10) concentration at all timepoints in control, strength and endurance groups after the Wingate test; *p < 0.05, **p < 0.01.

On the other hand, we observed an insignificant upward trend in the expression of the IL-10 marker in the control group between 6 h and 24 h after the Wingate test. The levels of this marker decreased significantly in endurance and strength groups while non-significantly in the control (Fig. 3). IL-10 is an anti-inflammatory cytokine that regulates the immune response. Its decrease indicates stress resulting from physical exertion, which, in the short term, may reduce an individual’s anti-inflammatory ability. We found no microbiome correlations with Il-10 in the control group after Wingate intervention.

#### Bruce treadmill test

When analyzing biochemical parameter differences after the Bruce intervention, we observed complex interactions, with the most pronounced adaptations to this type of effort occurring primarily in the endurance group (Table 2). The majority of correlations were linked to TIMP1 and related to amino acid metabolism, carbohydrate metabolism and bacterial biosynthesis. We identified 2 species and 2 pathways that were significantly associated with at least 2 blood markers: *Eubacterium rectale*, *Blautia wexlerae*; *PWY-5188: tetrapyrrole biosynthesis I from glutamate;* and *PWY-5030: L-histidine degradation III.* Notably, all of these markers were linked to TIMP1 and TfR, whereas *Eubacterium rectale* was additionally associated with Il-15.

There were also several associations within the control group. This time, however, most correlations were with LIF, particularly those involved in fatty acid biosynthesis, biotin biosynthesis, and amino acid metabolism. The *PWY0-1241: ADP-L-glycero-&beta;-D-manno-heptose biosynthesis* pathway was the microbiome parameter linked to multiple different blood markers: leptin, FSTL1 and SPARC.

**Table 2:** Spearman correlations of the Microbiome and parameter changes between timepoints 6 h and 24 h after Bruce intervention. Only correlations with significant p-values (< 0.05) are included.

Group	Parameter	Microbiome feature	Spearman correlation	Adjusted *p* value
Control	Leptin [pg/ml]	PWY0-1241: ADP-l-glycero-&beta;-d-manno-heptose biosynthesis	0.95	**< 0.001**
	LIF [pg/ml]	RIBOSYN2-PWY: flavin biosynthesis I (bacteria and plants)	0.90	**0.02**
	IL-6 [pg/ml]	Bacteroides cellulosilyticus	− 0.88	**0.03**
	LIF [pg/ml]	PWY-5971: palmitate biosynthesis (type II fatty acid synthase)	− 0.87	0.06
	Il-10 [pg/ml]	PWY0-1241: ADP-l-glycero-&beta;-d-manno-heptose biosynthesis	− 0.83	0.10
	LIF [pg/ml]	Bacteroides caccae	− 0.84	0.11
	LIF [pg/ml]	Clostridiaceae bacterium	0.80	0.12
	LIF [pg/ml]	BIOTIN-BIOSYNTHESIS-PWY: biotin biosynthesis I	− 0.80	0.12
	LIF [pg/ml]	FASYN-ELONG-PWY: fatty acid elongation—saturated	− 0.79	0.12
	LIF [pg/ml]	PWY-7664: oleate biosynthesis IV (anaerobic)	− 0.79	0.12
	LIF [pg/ml]	PWY-6609: adenine and adenosine salvage III	0.83	0.12
	LIF [pg/ml]	PWY-6519: 8-amino-7-oxononanoate biosynthesis I	− 0.79	0.12
	LIF [pg/ml]	PWY0-862: (5Z)-dodecenoate biosynthesis I	− 0.79	0.12
	LIF [pg/ml]	PWY-6282: palmitoleate biosynthesis I (from (5Z)-dodec-5-enoate)	− 0.79	0.12
	Leptin [pg/ml]	Roseburia faecis	− 0.82	0.15
	LIF [pg/ml]	PWY-6572: chondroitin sulfate degradation I (bacterial)	− 0.78	0.15
	FSTL1 [pg/ml]	PWY0-1241: ADP-l-glycero-&beta;-d-manno-heptose biosynthesis	− 0.80	0.16
	Il-10 [pg/ml]	PWY-6700: queuosine biosynthesis I (de novo)	0.80	0.17
	LIF [pg/ml]	PWY-8187: L-arginine degradation XIII (reductive Stickland reaction)	− 0.76	0.18
	SPARC [pg/ml]	PWY0-1241: ADP-l-glycero-&beta;-d-manno-heptose biosynthesis	− 0.80	0.18
Strength	IL-15 [pg/ml]	PWY0-1298: superpathway of pyrimidine deoxyribonucleosides degradation	− 0.95	**< 0.02**
	IL-10 [pg/ml]	Clostridium sp AF27 2AA	0.92	0.10
	IL-6 [pg/ml]	Gemmiger formicilis	0.92	0.11
Endurance	IL-15 [pg/ml]	Eubacterium rectale	− 0.95	**< 0.001**
	TfR [pg/ml]	Blautia wexlerae	− 0.93	**< 0.01**
	TIMP1 [pg/ml]	Bacteroides thetaiotaomicron	0.92	**< 0.01**
	TIMP1 [pg/ml]	PWY-5188: tetrapyrrole biosynthesis I (from glutamate)	− 0.92	**< 0.01**
	Adiponectin [pg/ml]	PWY3O-4107: NAD salvage pathway V (PNC V cycle)	− 0.92	**< 0.01**
	Adiponectin [pg/ml]	PWY-4041: &gamma;-glutamyl cycle	− 0.90	**< 0.01**
	TIMP1 [pg/ml]	Blautia wexlerae	− 0.90	**< 0.01**
	Adiponectin [pg/ml]	COLANSYN-PWY: colanic acid building blocks biosynthesis	− 0.89	**< 0.01**
	TIMP1 [pg/ml]	GLYCOGENSYNTH-PWY: glycogen biosynthesis I (from ADP-D-Glucose)	− 0.88	**< 0.01**
	TIMP1 [pg/ml]	PWY-5030: l-histidine degradation III	0.88	**< 0.01**
	TIMP1 [pg/ml]	HISDEG-PWY: l-histidine degradation I	0.88	**< 0.01**
	TIMP1 [pg/ml]	Odoribacter splanchnicus	− 0.88	**< 0.01**
	TIMP1 [pg/ml]	Bacteroides uniformis	0.84	**< 0.01**
	TIMP1 [pg/ml]	Dorea longicatena	0.84	**< 0.01**
	TIMP1 [pg/ml]	Lacrimispora celerecrescens	− 0.84	**< 0.01**
	TIMP1 [pg/ml]	OANTIGEN-PWY: O-antigen building blocks biosynthesis (*E. coli*)	− 0.84	**< 0.01**
	TIMP1 [pg/ml]	DAPLYSINESYN-PWY: l-lysine biosynthesis I	− 0.83	**< 0.01**
	TIMP1 [pg/ml]	CITRULBIO-PWY: L-citrulline biosynthesis	0.83	**< 0.01**
	TfR [pg/ml]	KETOGLUCONMET-PWY: ketogluconate metabolism	− 0.82	0.12
	TfR [pg/ml]	PWY-5188: tetrapyrrole biosynthesis I (from glutamate)	− 0.88	0.12
	TIMP1 [pg/ml]	PWY-7199: pyrimidine deoxyribonucleosides salvage	0.78	0.16
	TIMP1 [pg/ml]	NONOXIPENT-PWY: pentose phosphate pathway (nonoxidative branch) I	− 0.78	0.16
	TIMP1 [pg/ml]	Eubacterium rectale	− 0.78	0.16
	TIMP1 [pg/ml]	HSERMETANA-PWY: l-methionine biosynthesis III	− 0.78	0.16
	TIMP1 [pg/ml]	P441-PWY: superpathway of N-acetylneuraminate degradation	− 0.84	0.17
	TIMP1 [pg/ml]	ARGSYNBSUB-PWY: l-arginine biosynthesis II (acetyl cycle)	− 0.77	0.17
	TIMP1 [pg/ml]	METH-ACETATE-PWY: methanogenesis from acetate	− 0.77	0.17
	TIMP1 [pg/ml]	ARGSYN-PWY: l-arginine biosynthesis I (via l-ornithine)	− 0.77	0.17
	TfR [pg/ml]	PWY-5030: l-histidine degradation III	0.84	0.18
	TfR [pg/ml]	Eubacterium rectale	− 0.84	0.18
	TIMP1 [pg/ml]	PWY-7237: myo-, chiro- and scyllo-inositol degradation	− 0.76	0.19
	TIMP1 [pg/ml]	P41-PWY: pyruvate fermentation to acetate and (S)-lactate I	− 0.76	0.19
	TIMP1 [pg/ml]	PWY-6823: molybdopterin biosynthesis	− 0.76	0.19

TIMP1 (tissue inhibitor of metalloproteinases), IL-5 (interleukin 5), IL-6 (interleukin 6), IL-10 (interleukin 10), IL-15 (interleukin15), LIF (leukemia inhibitory factor), SPARC (secreted protein acidic and cysteine rich), TfR (transferrin receptor). Significant correlations (p-value < 0.05) were observed across all groups. In the control group, ADP-L-glycero-β-D-manno-heptose biosynthesis was positively correlated with leptin, while *Bacteroides cellulosilyticus* showed negative correlations with IL-6. The strength group revealed strong associations betweenof pyrimidine deoxyribonucleosides degradation and IL-15. In the endurance group, notable correlations included *Eubacterium rectale* with IL-15, and *Blautia wexlerae* with TfR, as well as various pathways involved in NAD (*N*icotinamide Adenine Dinucleotide) salvage and histidine degradation. These findings highlight the dynamic microbiome-blood marker interactions induced by the Bruce test across different exercise modalities.

Significant values are in bold.

**Fig. 4:**
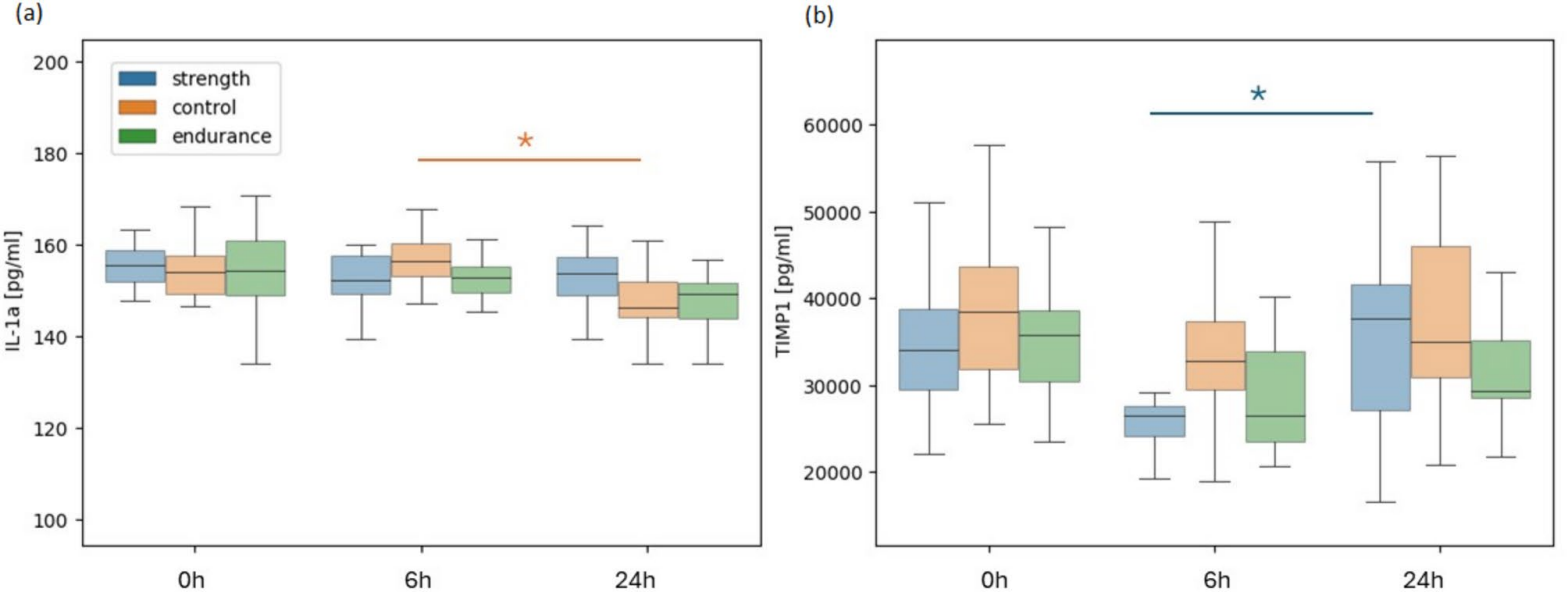
Differences in interleukin 1 alpha (IL-1a) and metalloproteinase inhibitor 1 (TIMP1) levels between training groups after the Bruce test. IL-1 A (**a**) and TIMP1 (**b**) trends after the Bruce test show a different response of the strength group. *p < 0,05.

Following the observation that the strength group had substantially fewer connections than the other groups did, we noticed a different trend in the two parameters, namely, IL-1a and TIMP1, in the strength group after the Bruce test (Fig. 4). The trend of IL-1a between 6 h and 24 h rapidly decreased in the control and endurance groups, whereas the mean value of the parameter in the strength group appeared to slightly increase. Similarly, TIMP1 expression increased rapidly between 6 h and 24 h in the strength group, whereas it appeared to be greater in the other two groups. Both IL-1a and TIMP1 are markers of the response to physical exertion. This decrease indicated the expected inflammatory behavior and initiation of body repair mechanisms. A different response in the strength group adapted to an anaerobic type of training (further referred to as the nonresponders), and control or endurance (further referred to as the responders) might have indicated greater stress and an altered response to aerobic exercise in the strength group.

### Baseline microbiome composition could be indicative of the adaptation response to aerobic intervention

**Fig. 5:**
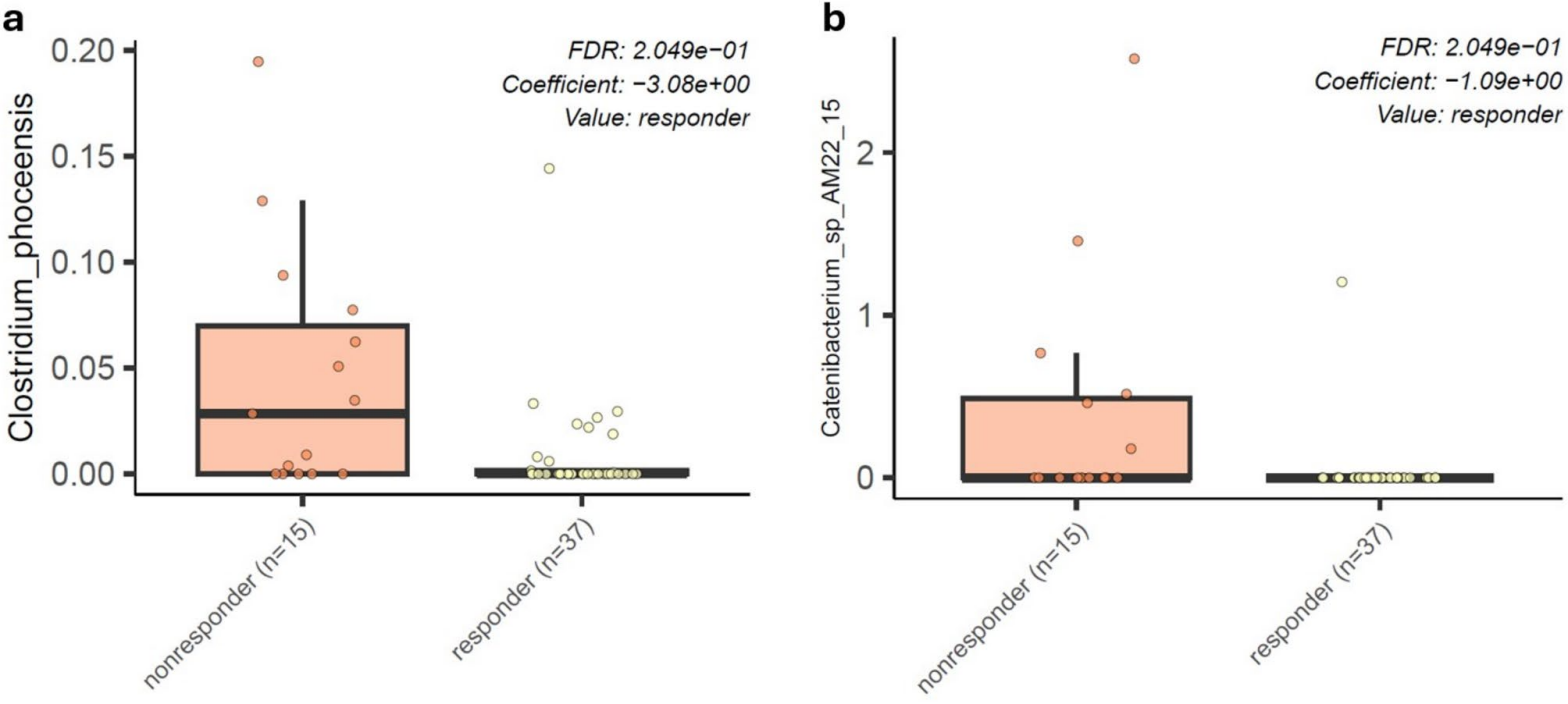
Baseline enrichment of specific bacterial species in Bruce test non-responders. Differential enrichment analysis identified two species that were more enriched in the baseline microbiome of the nonresponder group to the Bruce test.

We wondered whether the inhibited response to the Bruce intervention in the strength group could be associated with the microbiome composition at baseline before the intervention. Differential enrichment analysis with Maaslin2 between the strength group (nonresponders) and the control combined with the endurance group (responders) resulted in the identification of two species, *Clostridium phoceensis* and *Catenibacterium* spp. *AM22 15*, which were enriched in nonresponders versus responders at baseline before the intervention (Fig. 5). A function-based enrichment analysis identified pathways related to guanosine and adenosine biosynthesis as well as pyrimidine nucleobase salvage; however, the results did not pass false discovery correction.

## Discussion

Our study is one of the first to combine metagenomics and blood plasma markers to enhance the understanding of the role that the gut microbiome plays in recovery from a training intervention. Overall, our results reveal complex physiological and biochemical responses elicited by different exercise modalities and linked to training backgrounds. The multiomics approach, combined with a longitudinal study design, resulted in the identification of intergroup signals not discovered when investigating metagenomic data alone. In addition, our findings allowed us to hypothesize about potential mechanisms through which the gut microbiome modulates recovery from acute training interventions.

The changes in biochemical parameters following the Wingate and Bruce tests highlighted distinct physiological responses to different exercise modalities. Notably, these responses may also be shaped by the duration of each test protocol. The Wingate test is relatively short but extremely intense, potentially triggering rapid, acute stress responses. In contrast, the Bruce protocol is a progressive aerobic test that generally lasts longer and may produce a more sustained physiological challenge. As a result, biomarkers could exhibit different magnitudes or patterns of change, reflecting the varying metabolic demands and accumulated stress over time. Short, high-intensity efforts can lead to an immediate surge in certain inflammatory and metabolic markers that may subside quickly, whereas longer protocols may elicit both immediate and prolonged changes, thus influencing the 6–24-hour postexercise window differently. Changes appeared mostly between the 6th and 24th hours after the intervention. OSM levels significantly decreased across all groups postexercise, indicating a general reduction in inflammation, which is consistent with muscle regeneration processes^32^. Similarly, leptin levels decreased (significant in the strength group) after the Wingate test, reflecting the sensitivity of the hormone to anaerobic exercise and its role in energy regulation^33^. Follistatin-like 1 (FSTL1) showed a biphasic response, with an initial increase followed by a significant decrease, highlighting its involvement in inflammation and muscle repair after both types of exercise^34^. Markers such as LIF, resistin, IL-6 and TfR remained stable in both tests, indicating that these pathways were not significantly affected by the exercise protocols used.

SPARC and adiponectin were the only parameters that were conserved across control, strength and endurance but experienced different trends as a result of the two interventions. We noted connections of the markers with two pathways related to pyrimidine deoxyribonucleoside salvage and degradation. These pathways could potentially influence adiponectin levels through several indirect mechanisms. Metabolic pathways related to nucleotide degradation affect the levels of metabolites associated with oxidative stress and inflammation, which are known factors that lower adiponectin levels^35,36^. Nucleotides play a key role in cellular energy, and their degradation can affect energy availability, which in turn influences the ability of adiponectin to regulate energy metabolism and insulin sensitivity^37,38^. Additionally, nucleotide degradation products can affect cellular signaling, altering inflammatory and metabolic processes and impacting adiponectin levels^39,40^. Acute high-intensity exercise, such as the Wingate test, likely increases the demand for nucleotide catabolism, which is essential for DNA repair and energy production - critical processes during the recovery phase^41–44^. This prioritization of nucleotide metabolism could impact the synthesis or regulation of other proteins and hormones, including adiponectin, which is involved in lipid metabolism, insulin sensitivity, and anti-inflammatory responses^45^. The decreased adiponectin levels observed after the Wingate test may reflect a shift in metabolic priorities, favoring energy production and cellular repair processes^46^.

This divergent response of adiponectin could also be attributed to the different physiological demands and stress responses induced by the Wingate and Bruce tests. The Wingate test, which is a high-intensity anaerobic exercise, may induce a more pronounced acute inflammatory response, thereby affecting adiponectin levels more than the aerobic nature of the Bruce test, which typically induces a milder systemic response Additionally, it should be noted that the duration of the Bruce treadmill test was dependent on the participant’s aerobic fitness, leading to variability in inflammatory responses among individuals. This variability may influence adiponectin levels differently based on individual aerobic capacity and endurance. Consequently, the distinct physiological demands and stress responses elicited by the Wingate and Bruce treadmill tests likely contributed to the observed differences in inflammatory and metabolic responses. This observation suggests that the type and intensity of exercise (as well as potentially length) could play critical roles in modulating adiponectin levels and indicated the importance of considering exercise-specific responses in studies of metabolic and inflammatory markers^35,36,47^. The pyrimidine deoxyribonucleoside superpathway involves the degradation of pyrimidine nucleotides, which are essential components of DNA and RNA. The activity of this pathway may be linked to cellular and metabolic stress responses, potentially influencing systemic inflammation and metabolic regulation^35,39,40^. The strong negative correlation between this pathway and adiponectin suggests that higher pyrimidine catabolic activity may be associated with lower adiponectin levels. This could hypothetically be due to increased metabolic demands and cellular turnover during high-intensity exercise, such as the Wingate test, leading to potential changes in systemic markers such as adiponectin. It is possible that processes such as energy production and DNA repair are prioritized over the production of adiponectin in the immediate aftermath of exercise, possibly leading to the observed changes. Understanding these biochemical pathways and their interactions with metabolic markers may provide deeper insights into the molecular mechanisms underlying exercise-induced metabolic adaptations in different training groups^37^.

Following the Bruce treadmill test, an aerobic exercise protocol, we identified two bacterial species, *Eubacterium rectale* and *Blautia wexlerae*, and two metabolic pathways, *PWY-5188 (tetrapyrrole biosynthesis I from glutamate)* and *PWY-5030 (L-histidine degradation III)*, that were significantly associated with at least two blood markers. Notably, all of these markers were associated with TIMP1 (tissue inhibitor of metalloproteinases 1) and TfR (transferrin receptor), whereas *Eubacterium rectale* was additionally associated with IL-15. *Eubacterium rectale* and *Blautia wexlerae* are known as butyrate-producing bacteria that play critical roles in maintaining gut health and influencing systemic inflammation^49^. Butyrate has been shown to have anti-inflammatory properties and can modulate immune responses^50^, which may explain the associations of these bacteria with TIMP1 and TfR. TIMP1 is involved in tissue remodeling and has been linked to muscle recovery processes^51^, whereas TfR plays a role in iron metabolism, which is critical for oxygen transport and energy production during prolonged exercise^52^.

The association of *Eubacterium rectale* with IL-15, a cytokine involved in muscle maintenance and immune responses, could suggest a possible link between the gut microbiome and muscle physiology. IL-15 is known to be upregulated following endurance exercise and plays a role in promoting muscle growth and enhancing immune function^53^. IL-15 also seems to play a role in reducing adipose tissue mass^53^. The concept of a gut-muscle axis, where the microbiome plays a role in exercise-induced muscle adaptation, has been supported by multiple animal and human studies^8,54,55^. Our finding suggests that *Eubacterium rectale* may contribute to this axis, potentially influencing muscle physiology through its interaction with IL-15. Further research is needed to determine whether *E. rectale* directly modulates IL-15 expression or if their association is driven by broader host-microbiome interactions. Understanding this relationship could provide new insights into how gut microbiota influence exercise performance, recovery, and metabolic health.

The *tetrapyrrole biosynthesis pathway (PWY-5188)* and *L-histidine degradation pathway (PWY-5030)* provide further insight into the metabolic changes that occur in response to endurance exercise. The tetrapyrrole biosynthesis pathway is crucial for the production of heme, which is necessary for oxygen transport and mitochondrial respiration - processes that are particularly important during sustained aerobic exercise^56^. The L-histidine catabolic pathway involves the breakdown of histidine, which can affect histamine production and ammonia detoxification. Both processes are relevant in the context of prolonged exercise, where histamine is involved in vasodilation and the immune response, and ammonia detoxification is critical for preventing metabolic acidosis^57^. These findings suggest that the specific gut microbiome and metabolic pathways associated with endurance exercise may contribute to the modulation of physiological adaptations and recovery processes through their effects on inflammation, iron metabolism, and energy production. Further research is needed to understand the precise mechanisms by which these bacteria and pathways interact with host physiology and to explore potential interventions that could enhance recovery and performance in endurance exercise.

Finally, two species—*Clostridium phoceensis* and *Catenibacterium* spp. AM22 15—were identified as being enriched in individuals who exhibited an inhibited response (strength group) to the Bruce intervention at baseline. Our previous work demonstrated that differences in physiological markers, such as mean and maximal power, did not always align with the selected training regimen (Supplementary Table S1). The inhibited response to aerobic intervention in the strength group was one of the few findings that clearly distinguished this group from the others. Given this, we were confident in categorizing individuals as responders or non-responders in a binary manner and proceeded with a differential enrichment analysis to further distinguish the two groups. *Clostridium phoceensis* contains machinery to synthesize tryptophan and has previously been shown to respond to acute moderate-intensity exercise intervention^58^. Tryptophan metabolism is known to play a role in immune regulation and inflammation. Alterations in tryptophan metabolism can affect the production of metabolites such as kynurenine, which have been implicated in the modulation of inflammation and immune responses^59^. The presence of *Clostridium phoceensis*, which has tryptophan-synthesizing capabilities, may suggest a distinct tryptophan metabolic potential in the strength group, although further studies measuring kynurenine and related metabolites are needed to determine a direct link to inflammatory and stress responses. *Catenibacterium* spp. *AM22 15* was enriched in the strength group at baseline before the Bruce intervention in our previous study^23^. The enrichment of these species may also contribute to the distinct metabolic and inflammatory patterns observed in the strength group. The distinct microbiome composition in the strength group, characterized by these specific bacterial species, may help explain the group’s inhibited response to Bruce intervention, although further investigation is required to clarify the interplay between microbiome composition, tryptophan metabolism, and exercise response.

The greatest limitation of this study is its small sample size. As discussed in our previous publication, despite the participants being athletic or untrained, the overall group was quite homogeneous. Furthermore, in addition to sample size, the duration of the exercise protocols may influence the generalizability of the findings. Short, high-intensity protocols and longer, progressive tests each create distinct metabolic and physiological stress patterns, which could yield different biomarker profiles and microbiome shifts. Future studies should consider standardizing or systematically varying the duration of exercise tasks to account for these differences in stress exposure and better capture the dynamic interplay between exercise duration, intensity, and the observed biochemical or microbial changes. Although participants were instructed to maintain their usual dietary habits before and throughout the study, no formal dietary tracking or nutritional analysis was conducted. Further studies with larger sample sizes and formal tracking of lifestyle factors such as diet are needed to validate the results reported in this manuscript.

## Conclusions

This study highlights the complex interplay between exercise modality, training background, and the gut microbiome in shaping physiological responses. By integrating metagenomic data with blood serum marker data, we observed that both aerobic and anaerobic exercise induce specific biochemical changes that may be influenced by the gut microbiome.

Key findings include distinct responses of SPARC and adiponectin levels depending on exercise modality, suggesting that these markers are differentially modulated by aerobic and anaerobic efforts. In strength-trained individuals, unique associations between specific gut microbes and blood markers were observed after the anaerobic Wingate test, suggesting that training background may influence microbiome-mediated responses to exercise. Conversely, endurance athletes showed correlations between butyrate-producing bacteria and markers related to muscle recovery and iron metabolism after the aerobic Bruce test, suggesting a potential gut‒muscle axis.

A baseline enrichment of certain bacterial species, such as *Clostridium phoceensis* and *Catenibacterium* spp., in strength athletes was associated with an inhibited response to the aerobic intervention. These findings suggest that the initial microbiome composition may influence how individuals adapt to different types of exercise. However, further validation in larger, diverse cohorts is needed to confirm these associations and to better understand the underlying mechanisms.

These findings, while requiring further validation, highlight the important role the gut microbiome may play in modulating exercise-induced physiological adaptations. Longitudinal studies and mechanistic investigations will be essential to determine whether these microbial signatures have a causal role in exercise adaptation or simply reflect pre-existing physiological differences among individuals. Understanding these relationships could lead to personalized microbiome-targeted interventions to enhance athletic performance and recovery on the basis of individual training backgrounds.

## Electronic supplementary material

Below is the link to the electronic supplementary material.

Supplementary Material 1

## Data Availability

The data supporting the findings of this study are openly available in the European Nucleotide Archive (ENA) at https://www.ebi.ac.uk/ena/, reference number PRJEB60692.
