# Supplementary material for: Gut microbiome and blood biomarkers reveal differential responses to aerobic and anaerobic exercise in collegiate men of diverse training backgrounds
Source: Sci Rep. 2025 May 8;15:16061. doi: 10.1038/s41598-025-99485-9 (PMC12062308; doi:10.1038/s41598-025-99485-9)
Supplement: Supplementary file 1 — Supplementary Material 1 [file 41598_2025_99485_MOESM1_ESM.docx]

Supplementary Information

Gut microbiome and blood biomarkers reveal differential responses to aerobic and anaerobic exercise in collegiate men of diverse training backgrounds

Kinga Humińska-Lisowska^1*^, Monika Michałowska-Sawczyn^1^, Tomasz Kosciolek^2^, Paweł P. Łabaj^3^, Andrzej Kochanowicz^4^, Jan Mieszkowski^4^, Barkin Bicakci^1^, Patrizia Proia^5^, Paweł Cięszczyk^1^, Kinga Zielińska^3^*

1 - Faculty of Physical Culture, Gdansk University of Physical Education and Sport, Gdansk, Poland

2 - Sano Centre for Computational Medicine, Krakow, Poland

3 – Malopolska Centre of Biotechnology, Jagiellonian University, Krakow, Poland

4 - Faculty of Health Sciences, University of Lomza, Lomza, Poland

5 - Sport and Exercise Sciences Research Unit, Department of Psychology, Educational Science and Human Movement, University of Palermo, Palermo, Italy

Correspondence:

* Kinga Humińska-Lisowska, kinga.huminska-lisowska@awf.gda.pl

Supplementary Table S1. Fitness parameter values in each study group.

| **Variable** | **Intervention** | **Unit** | **Control (n=21)    Mean +- std** | **Strength (n=16)   Mean +- std** | **Endurance**  **(n=15)**  **mean +- std** | **ANOVA (p-value, η^2^)** |
| --- | --- | --- | --- | --- | --- | --- |
| **VO_2_max** | Bruce | ml/min/kg | 55.6 +- 5.9 | 58.4 +- 7.2 | 61.3 +- 7.6* | 0.07, 0.12 |
| **Relative mean power** | Wingate | W/kg | 8.0 +- 0.7 | 8.4 +- 0.7 | 8.2 +- 0.6 | 0.11, 0.09 |
| **Relative peak power** | Wingate | W/kg | 10.0 +- 1.0 | 10.8 +- 1.0 | 10.1 +- 1.1 | 0.07, 0.11 |
| **Relative mean power MAINTAINED** | Wingate | W/kg | 0.7 +- 0.1 | 0.7 +- 0.0 | 0.7 +- 0.1 | 0.33, 0.05 |
| **Relative peak power MAINTAINED** | Wingate | W/kg | 0.8 +- 0.1 | 0.7 +- 0.1 | 0.8 +- 0.1 | 0.10, 010 |

*statistically significant differences from the control group (t-test, p<0.05). Relative mean and peak power maintenance refer to the proportion of power sustained in the second Wingate effort compared to the first. VO_2_max differences were observed, with the endurance group showing significantly higher values compared to the strength and control groups. For other parameters, no significant between-group differences were detected, which may reflect overlapping physiological adaptations across training backgrounds or inter-individual variability typical in exercise response.

Supplementary Table S2. Post-exercise changes in biochemical markers in all groups following the Wingate and Bruce treadmill tests – further statistics.

| Intervention | Parameter | Group | Cohen’s d (effect size) | Adjusted p-value |
| --- | --- | --- | --- | --- |
| Wingate | TIMP1 [pg/ml] | Strength | -1.25 | < 0.001 |
|  |  | Endurance | -1.05 | 0.012 |
|  |  | Control | -1.22 | 0.004 |
|  | Il-10 [pg/ml] | Strength | 0.72 | 0.007 |
|  |  | Endurance | 1.64 | 0.005 |
|  | SPARC [pg/ml] | Control | -0.95 | 0.001 |
|  | FSTL1 [pg/ml] | Strength | 0.98 | 0.007 |
|  | IL-15 [pg/ml] | Strength | 1.42 | < 0.001 |
|  |  | Endurance | 1.31 | < 0.001 |
|  |  | Control | 1.56 | < 0.001 |
|  | Adiponectin [pg/ml] | Strength | 1.06 | 0.001 |
|  |  | Endurance | 1.08 | 0.001 |
|  |  | Control | 1.10 | 0.003 |
|  | IL-1a [pg/ml] | Strength | 1.11 | 0.020 |
|  | Oncostatin [pg/ml] | Strength | 1.44 | < 0.001 |
|  |  | Endurance | 1.98 | 0.002 |
|  |  | Control | 1.71 | < 0.001 |
|  | Leptin [pg/ml] | Strength | 2.32 | < 0.001 |
|  |  | Endurance | 0.73 | < 0.001 |
|  |  | Control | 0.53 | < 0.001 |
| Bruce | TIMP1 [pg/ml] | Strength | -1.11 | 0.025 |
|  | FSTL1 [pg/ml] | Strength | 1.03 | 0.008 |
|  |  | Endurance | 1.11 | 0.008 |
|  |  | Control | 0.91 | 0.016 |
|  | IL-15 [pg/ml] | Strength | 0.54 | 0.004 |
|  |  | Endurance | 1.08 | 0.007 |
|  |  | Control | 0.85 | < 0.001 |
|  | IL-1a [pg/ml] | Endurance | 0.65 | 0.013 |
|  |  | Control | 0.95 | 0.005 |
|  | Oncostatin [pg/ml] | Strength | 2.02 | < 0.001 |
|  |  | Endurance | 1.42 | < 0.001 |
|  |  | Control | 1.00 | 0.026 |
|  | Leptin [pg/ml] | Endurance | 0.87 | 0.018 |
|  |  | Control | 0.34 | 0.002 |

Metalloproteinase inhibitor 1 (TIMP1), interleukin (IL)-1 alpha, IL-6, IL-10, IL-15, oncostatin M (OSM), follistatin-related protein 1 (FSTL1), secreted protein acidic and rich in cysteine (SPARC).

Significant changes were observed in various markers, with the strength and endurance groups showing notable differences in IL-15, leptin, and oncostatin, particularly after the Wingate test, which induces more immediate physiological responses. The Bruce test also resulted in significant alterations, typically in markers related to aerobic metabolism and longer-term physiological adaptation. High effect sizes and significant p-values underscore the distinct impacts of these exercises on biochemical parameters, with the Wingate test eliciting more short-term, high-intensity responses, and the Bruce treadmill test reflecting more gradual, endurance-related adaptations.
